# Coronary Physiology: Modern Concepts for the Guidance of Percutaneous Coronary Interventions and Medical Therapy

**DOI:** 10.3390/jcm12062274

**Published:** 2023-03-15

**Authors:** Monica Verdoia, Andrea Rognoni

**Affiliations:** Nuovo Ospedale Degli Infermi, Azienda Sanitaria Locale Biella, 13900 Biella, Italy

**Keywords:** coronary flow, coronary reserve, percutaneous coronary intervention

## Abstract

Recent evidence on ischemia, rather than coronary artery disease (CAD), representing a major determinant of outcomes, has led to a progressive shift in the management of patients with ischemic heart disease. According to most recent guidelines, myocardial revascularization strategies based on anatomical findings should be progressively abandoned in favor of functional criteria for the guidance of PCI. Thus, emerging importance has been assigned to the assessment of coronary physiology in order to determine the ischemic significance of coronary stenoses. However, despite several indexes and tools that have been developed so far, the existence of technical and clinical conditions potentially biasing the functional evaluation of the coronary tree still cause debates regarding the strategy of choice. The present review provides an overview of the available methods and the most recent acquirements for the invasive assessment of ischemia, focusing on the most widely available indexes, fractional flow reserve (FFR) and instant-wave free ratio (iFR), in addition to emerging examples, as new approaches to coronary flow reserve (CFR) and microvascular resistance, aiming at promoting the knowledge and application of those “full physiology” principles, which are generally advocated to allow a tailored treatment and the achievement of the largest prognostic benefits.

## 1. Background

The improvements in percutaneous coronary intervention (PCI) techniques and the availability of newer generations of drug-eluting stents (DES) have led to an escalation in the number and complexity of procedures [1,2,3]. Nevertheless, impaired outcome has been observed in certain subsets of patients experiencing higher rates of target lesion failure and recurrent ischemic events [4,5], as well as bleeding complications in consequence of the antithrombotic agents. Therefore, a new approach of limiting PCI procedures and stent implantation only when clearly required has become the most common practice among interventionalists. In fact, optimal medical therapy has been shown to be superior to an interventional strategy in particular conditions, thus pointing to the need for non-anatomical criteria to be considered for the guidance of PCI procedures.

Indeed, angiographic criteria for the quantification of the severity of a lesion have been proven unsatisfactory for the correct stratification of its risk of instabilization and functional impact [6,7]. In fact, other parameters, in addition to the conventional minimal lumen diameter and the percentage of stenosis at angiography, have been shown to play a relevant prognostic role. Lesion length, plaque morphology, amount of served myocardium and distal vascular bed, in addition to the complex regulation of vasomotricity, represent factors that should certainly be taken into account, although requiring further diagnostic investigation after angiography, by the use of intracoronary imaging or functional tests [8,9].

The invasive assessment of the ischemic potential of coronary lesions has gained ground due to accumulating of clinical evidence, currently being recommended as a class Ia strategy for the management of intermediate coronary lesions, according to European guidelines, and as a class IIa strategy, according to the U.S. regulations [10,11].

Several indexes of coronary physiology have been proposed, whose features are shown in Table 1, although these are affected by technical and clinical factors; therefore, these indexes may provide potentially incomplete or misleading results. Moreover, acute patients and particular coronary anatomies, such as tandem lesions, as well as diffuse and microvascular disease, still represent particularly challenging settings, in which the assessment of coronary physiology is still poorly validated.

## 2. Traditional Indexes: Fractional Flow Reserve

Fractional flow reserve (FFR) was introduced around 20 years ago in order to provide a faster and easier estimation of the hemodynamic impact of a stenosis on the coronary reserve, based on the assumption that coronary pressure is proportional to coronary flow, when coronary resistance is minimal and constant [12].

In fact, the FFR is the ratio between mean coronary pressure distal to a coronary stenosis and mean aortic pressure at the level of the guiding catheter measured during maximal pharmacological vasodilation, when resistance is considered to be null.

Two randomized clinical trials have demonstrated the safety and effectiveness of conditioning coronary revascularization, according to the results of FFR.

The DEFER study [13] randomized patients with stable angina and intermediate stenosis, but FFR > 0.75, to deferral or performance of PCI. The authors concluded that patients with FFR > 0.75 were stable and safe, and that stenting did not decrease the risk of cardiac events for CAD without significant ischemia.

The FAME study [14] compared FFR-guided PCI with angio-guided PCI in patients with multivessel CAD, showing a significant reduction of MACE at 1 year with FFR. In this study, PCI was indicated for FFR values < 0.80, and around 30% of patients presented with acute coronary syndrome (ACS), although no dedicated sub-analysis was performed.

Similar advantages were confirmed in the subsequent FAME2 trial [15], where FFR-guided PCI was compared with optimal medical therapy (OMT), which was interrupted for excess benefit with PCI, mainly driven by a reduction in recurrent revascularization (HR 0.13; 95% CI 0.06–0.30, *p* < 0.001).

Indeed, only 1047 patients were randomized to FFR within the three trials; however, subsequent studies and a recent Japanese multicenter registry confirmed the safety of relying on FFR, leading to a reclassification and “downgrading” of the functional impact of a stenosis in around 40% of the cases [16].

However, discrepancy between FFR and coronary flow reserve has been documented in around 30% of the patients [17], mainly due to technical and clinical factors, conditioning the hemodynamic status and therefore, the assessment of FFR.

In fact, the invasively validated measurement of FFR, derived from an experimental animal model, was corrected for right atrial pressure, whose omission, in the clinically used “crude” FFR, could lead to falsely higher values and thus, to an underestimation of the significance of a lesion, especially in subjects with elevated diastolic pressures, thus accounting for the elevation of the cut-off to 0.80 [18].

**Figure 1:**
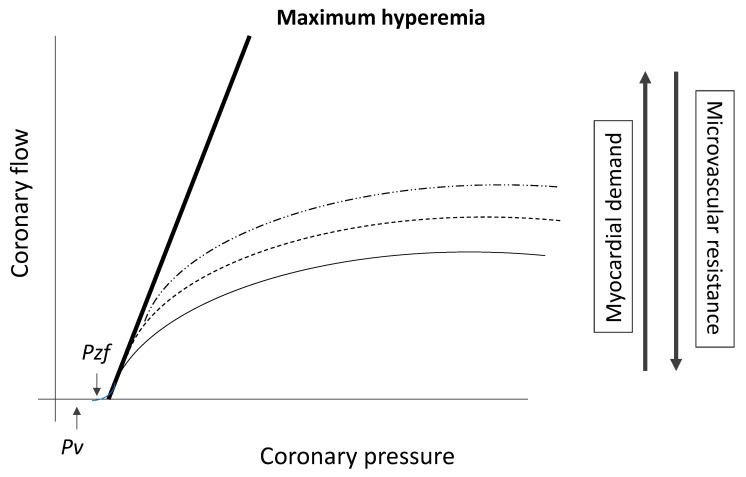
Relationship between coronary blood flow and pressure during different hemodynamic conditions (Pv = venous pressure; Pzf = baseline pressure corresponding to zero-flow condition), adapted from van de Hoef et al. [19].

In effect, even if constant coronary resistance could be achieved, the relationship between pressure and flow in the coronary circulation is not linear, but incremental, with a variable slope and a baseline intercept which should correspond to coronary wedge pressure ([19], Figure 1).

Moreover, the complete abolition of coronary resistance, i.e., the achievement of maximal hyperemia, represents a debated issue. Among hyperemic agents, for years, adenosine has represented the pharmacological strategy of choice, although weighted by a longer duration of the exam, complications, patients’ intolerance, especially with intravenous infusion of adenosine, as well as inadequate response, being more common with low-dose adenosine boli and among certain subsets of patients [20].

In fact, previous studies suggested an age-related decrease in absolute coronary vasodilator reserve, mediated by a lower response to endothelium-derived relaxing factors in a dysfunctional endothelium, but also by a lesser effect of adenosine on vascular smooth muscle due to desensitization. In fact, experimental models have shown a greater local release of adenosine in the coronary microcirculation of older animals, which could potentially impair the effectiveness of an exogenous administration [21].

Nevertheless, alternative pharmacological agents for the achievement of hyperemia, such as papaverine, have not been validated in large scale studies [22].

## 3. Non-Hyperemic Indexes: Instant-Wave Free Ratio (iFR)

The stability of coronary flow across a wide range of coronary stenoses under baseline resting conditions provides an ideal setting for the assessment of coronary lesions through pressure-derived indexes.

The identification of a wave-free period during the diastolic period of the cardiac cycle (from 25% of the initiation of the diastole to 5 ms before its ending), where coronary flow is maximal and myocardial contraction is absent, represents a condition of resting “maximal” coronary flow that can be assimilated to the hyperemic condition, although not requiring pharmacological vasodilatation [23].

The instant wave-free flow reserve (iFR) is, therefore, defined as the ratio between the mean instant pressure distal to coronary stenosis and the mean pressure at the aortic root during such periods, as identified on the electrocardiogram. The ADVISE (Adenosine Vasodilator Independent Stenosis Evaluation) and ADVISE Registry studies were the first to assess the diagnostic accuracy of iFR against FFR as the ischemic reference standard [24,25]. It emerged that iFR had a good correlation with FFR (r = 0.9, *p* < 0.001) and showed excellent diagnostic efficiency, with fewer repeated measurement differences.

The JUSTIFY-CFR study showed that iFR had a better correlation with coronary flow reserve than did FFR [26]. Moreover, van de Hoef et al. compared iFR and FFR, with reference to myocardial perfusion scintigraphy. They found that iFR and FFR were discordant in around 20% of the cases, but FFR did not always produce highly accurate results, and thus, was not necessarily a better discriminator of coronary ischemia than iFR [27].

The subsequent large-scale trials DEFINE-FLAIR [28] and iFR SWEDEHEART [29] randomized over 4500 patients to show the noninferiority of iFR to FFR. The two studies and the subsequent pooled analysis confirmed the safety of deferring myocardial revascularization according to coronary physiology in patients with intermediate-severity coronary lesions, and the noninferiority of an iFR- versus a FFR-guided strategy, additionally pointing at the potential advantages of the non-hyperemic index in terms of procedure duration, patients’ tolerance, and complications [23,30].

Theferore, iFR has been equated to FFR in most recent 2019 ESC guidelines on myocardial revascularization [31].

Indeed, iFR has been suggested to be more sensitive to hemodynamic variations in blood pressure and heart rate, conditioning the duration of diastolic period, than FFR. Moreover, patients in atrial fibrillation could present another limitation of the diagnostic accuracy of iFR, due to the instant estimation of the value and the larger variability of the diastolic period, which could be instead balanced by an averaged measure as FFR.

In a previous study, Verdoia et al. demonstrated that pre-procedural beta-blockers were associated with higher absolute values and a lower rate of positive iFR, as compared to FFR [32]. Nevertheless, contrasting results were demonstrated by the same authors in elderly patients, where FFR levels tended to be higher and potentially underestimative, whereas iFR was less affected by age [33].

Other potential advantages of iFR are linked to technical factors that can potentially overcome the limitations of FFR. In fact, FFR is more sensitive to catheter dumping or wire-drift, the latter often being dependent on the entrapment of small air bubbles in the cavity of the pressure sensor or electrical and thermal instability, which can occur more frequently in longer registrations as in the FFR [34,35]; FFR has been demonstrated to be largely inadequate in the presence of proximal stenosis, reduced myocardial viability, or modest distal territory, or in the case of elevated diastolic pressure and microvascular disfunction, conditions that occur quite frequently in patients with advanced age, hypertension, diabetes, renal failure, or ACS, representing the majority of current cath-lab population [36].

Similar limitations have been observed in low-flow conditions, such as aortic stenosis or left ventricular dysfunction, inducing peripheral vasoconstriction and an increase in microvascular resistances in order to maintain the perfusion, causing an increase in the distal pressure (Pd) and a reduction of proximal pressure (Pa), thus resulting in potentially overestimated FFR values.

In addition, the avoidance of pharmacological hyperemia with iFR has shown more promising results in terms of safety, and mainly among patients with acute coronary syndrome. In the COMPARE-ACUTE trial, which assessed FFR guided revascularization versus conventional strategy in acute STEMI patients with multivessel disease, serious adverse events occurred in 0.2% of the study population, including dissections and abrupt vessel occlusion, with negative prognostic consequences [37].

Indeed, only around 15% of the patients had ACS at presentation in the main iFR trials; however, iFR results led to similar outcomes, regardless of clinical presentation [38], and similar results were also confirmed in large scale registries [39].

Moreover, iFR could be less conditioned by the impairment of microvascular circulation, which often occurs in patients with ACS, and especially in STEMI settings, where peripheral resistances progressively reduce within days, allowing for a proportional increase in flow [40].

Another scenario in which iFR has been suggested to overcome FFR is represented by complex tandem lesions and diffuse disease. In fact, the presence of a proximal stenosis could result in a decreased coronary pressure, leading to an underestimation of the distal stenosis, and inversely, the distal obstruction could raise the Pd, resulting in lower FFR values.

The instantaneity of the iFR, allowing a point-by-point measurement alongside a coronary vessel, without time restrictions linked to the duration of hyperemia, provides the possibility of separately interrogating different parts of a diseased vessel, with the pullback of the wire, not being additionally conditioned by pressure interactions for lesions in series [41].

**Figure 2:**
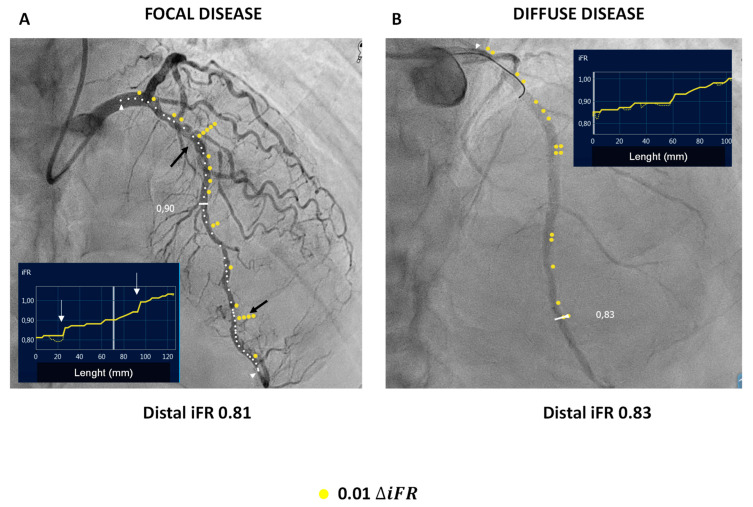
Co-registration of pullback instant wave-free flow reserve (iFR) and coronary angiography. (**A**) (left image) displays a pattern of focal disease: two pressure drops are evident in the iFR registration graph (white arrows) corresponding to two focal stenoses on the mid and distal left anterior descending artery (black arrows). (**B**) (right image) shows a pattern of diffuse disease: no significant pressure drop is evident in the iFR graph.

Moreover, motorized iFR pullback recording, combined with real-time computer tracking of the pressure-wire movement, provides a complete physiological map of any coronary vessel, whereby the graphical representation of the point estimations of the iFR can be integrated with a co-registered angiogram, providing a fusion of functional and anatomical information [42], as show in Figure 2.

The development of such technology allows for instant calculation of the predicted post-PCI iFR values, thus potentially allowing for the minimizing of interventional approaches to only significant lesions, especially in diffuse multivessel disease.

Moreover, the implementation of iFR with co-registration could even overcome the potential advantages of quantitative flow ratio (QFR), a computational model based on blood flow velocity and calculated on three-dimensional coronary tree images, which are acquired with coronary CT or angiography [43,44]. QFR allows for attributing a physiological significance to pure anatomical findings, although representing only a mathematical estimation, thus far less precise than a direct measurement, as in iFR.

## 4. From Stenosis to Ischemia: New Concepts of Coronary Physiology

Pressure indexes have been used for the assessment of coronary stenoses for an extended period of, based on the demonstration of a direct association between coronary pressure and flow.

However, the relationship between coronary pressure and flow is not linear, but incremental, with a variable slope (Figure 1) and a non-zero intercept, being largely conditioned by the variations in coronary resistances, which are increased in stenosed as compared to healthy coronary arteries [19].

Thus, coronary pressure evaluation only provides a crude and largely imprecise estimation of the impairment of coronary flow, resulting in the underestimation of the disease, especially in conditions of generalized low flow and in particular, in the absence of angiographically apparent epicardial stenoses. Thus, low coronary flow may be observed, despite a normal FFR value, whereas an abnormal FFR value can coexist with preserved coronary flow, leaving myocardial function unaffected down to FFR values of around 0.4 [45].

Microvascular coronary circulation plays a key role in such processes, representing the principal site of regulation of coronary resistances. In fact, in healthy coronary vessels, the epicardial arteries only account for up to 10% of coronary resistance, while the development of atherosclerotic lesions can significantly increase such levels of resistance, counterbalanced by a progressive adaptative reduction in the resistance of arterioles, which undergo autoregulatory vasodilation. Reaching a complete exhaustion of the reserve vasodilatory capacity can lead to myocardial ischemia. Indeed, as pointed out in recent studies, the presence of ischemia exhibits a greater prognostic weight, independently from the existence of epicardial stenoses.

Despite the dominant role of coronary microcirculation in myocardial perfusion, it has been largely neglected by currently available methods for the assessment of coronary physiology, mainly due to the technical complexity of its evaluation.

However, several factors can lead to an impairment of coronary microcirculation, even in the absence of atherosclerotic obstructions, such as left ventricular hypertrophy, hypertension, diabetes, and aging itself, conditions that are becoming more and more common, especially with the progressive increase in elderly patients admitted to the cath lab.

Thus, increasing attention has been focused on the evaluation of microvascular functional status in clinical settings.

In addition to non-invasive provocative tests that cannot be performed during angiography, but must be either obtained in advance or re-accessed during invasive procedures, flow assessment can be performed during coronary angiography by thermodilution or the Doppler technique, both of which provide a measure of coronary flow velocity. The thermodilution-derived CFR has been validated in animal and human models and has been compared in an animal model to a reference standard of absolute flow, appearing to correlate more closely to the standard than does Doppler-derived CFR [46].

Flow velocity in the coronary circulation is only moderately reduced at every bifurcation, due to the reduction of the diameter of vessels in the arterial bed downstream, in consequence of the increase in total cross-sectional area, which is directly related to the amount of perfused myocardial mass [47].

Coronary flow reserve (CFR), the ratio of coronary flow velocity during maximal vasodilation to coronary flow velocity during resting conditions, is a widely studied and well-validated flow-based physiological parameter which has recently been successfully applied to coronary invasive assessment modalities, with the development of a wire-based thermodilution technique [48,49].

The pressure sensor can also act as a thermistor. With commercially available software, the shaft of the wire acts as a proximal thermistor. Room temperature saline can be injected into the coronary artery, and this system will calculate the transit time, which is inversely proportional to coronary flow. Measurements after 3 mL of saline injection per time are repeated, both at rest and after the induction of hyperemia, which can be achieved with several agents (papaverine, nitroprusside, or adenosine), although the latter, with intravenous infusion, currently represents the most applied agent [50].

**Figure 3:**
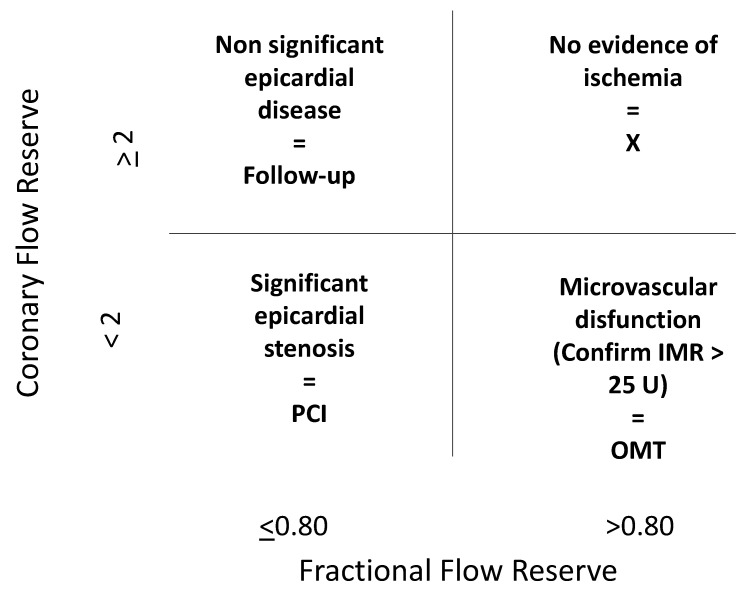
Diagnostic and clinical implications of a “full physiology” approach based on the relationship between fractional flow reserve and coronary flow velocity reserve.

Both requiring the achievement of hyperemia, CFR and FFR are nevertheless extremely different, interrogating different domains of the coronary tree, the latter being more focused on epicardial vessels, while the former on microvasculature. In fact, large-scale application of CFR, in addition to pressure-indexes, has led to the evidence of discordance between FFR and CFR in 30–40% of vessels with intermediate stenoses. As recently well shown by van de Hoef et al. [19] and depicted in Figure 3, abnormal fractional flow reserve and a normal CFR should indicate epicardial disease, while not flow-limiting, whereas normal FFR and a CFR < 2.0 should represent a predominant microvascular involvement.

Nevertheless, a variability in the normality of CFR has been reported, with a range between approximately 2.5 and 6; therefore, a value >2.0 can be abnormal for a particular patient in a specific vessel. For example, Demir et al. recently documented an overestimation of thermodilution-derived CFR as compared to Doppler-derived CFR, with a poor diagnostic accuracy of the common threshold of <2.0 and a weak correlation between hyperemic microvascular resistance and the index of microvascular resistance [51]

Furthermore, few randomized trials have so far addressed the prognostic impact of CFR.

The DEFINE-FLOW trial [52] randomized 430 patients with a positive FFR to undergo or postpone PCI according to CFR values (< or ≥2.0). The trial reported an increase in myocardial revascularization and infarction when deferring PCI in hemodynamically significant lesions with preserved CFR. However, according to the investigators, the unblinded design may have also driven the performance of interventions in the deferred arm.

On the contrary, opposite positive results have emerged when applying CFR to patients with angina symptoms and/or signs of ischemia, but no obstructive coronary artery disease (INOCA). In fact, rather than PCI guidance, the evaluation of microvascular physiology, in association with traditional pressure indexes, in patients with ischemic heart disease (IHD) is currently becoming the major field of application for CFR.

## 5. Coronary Physiology in Patients with INOCA

Among patients presenting with evidence of acute or chronic ischemia, the absence of epicardial coronary stenoses is currently becoming more frequent, being observed in up to 50% of the patients undergoing coronary angiography, even with a positive noninvasive test [53].

Coronary microvascular disfunction has been claimed for such findings, although still representing a challenging condition, whose identification and management is largely undefined.

However, several studies and a recent large-scale meta-analysis [54] have well defined the negative prognostic role of myocardial ischemia, even in the absence of macroscopic coronary artery disease. In Kelshiker et al. [54], abnormal coronary flow reserve (CFR) was associated with a higher incidence of all-cause mortality (HR: 3.78, 95% confidence interval (CI): 2.39–5.97) and a higher incidence of MACE (HR 3.42, 95% CI: 2.92–3.99), with a proportional increase in mortality of 16% per a 0.1 CFR unit reduction. These data were also confirmed for mortality (HR: 5.44, 95% CI: 3.78–7.83) and MACE (HR: 3.56, 95% CI: 2.14–5.90) among patients with isolated coronary microvascular dysfunction.

Thus, novel indexes, specifically addressing the identification of impairments of microvasculature, have been progressively developed.

The index of microvascular resistance (IMR) is a parameter specifically addressing microvasculature, which can be derived from thermodilution-derived mean transit time during CFR measurement. An IMR greater than 25 units is considered abnormal and diagnostic for microvascular angina [55].

In the CorMicA Trial, among 391 patients with angina, a stratified medical therapy driven by coronary function testing resulted in an improvement in quality of life, although not in conditioning long-term outcomes [56].

In effect, the relevant benefits of optimizing medical therapy, independent of coronary revascularization, have recently emerged in several randomized trials [57,58].

Nevertheless, the pharmacological agents preferred in patients with INOCA are still poorly defined in the absence of dedicated randomized trials.

In the retrospective analysis of a large cohort of patients with acute coronary syndrome and no evidence of coronary epicardial disease, Ciliberti et al. [59] documented that the use of beta-blockers was significantly associated with a less frequent occurrence of adverse outcomes at long-term follow-up among patients with MINOCA, whereas ASA displayed a potentially harmful impact on prognosis.

Similar conclusion was reached by Lindahl et al. in a subanalysis of 9138 patients with MINOCA enrolled in the SWEDEHEART registry (the Swedish Web-System for Enhancement and Development of Evidence-Based Care in Heart Disease Evaluated According to Recommended Therapy). After a mean follow-up of 4.1 years, there was a significantly lower event rate associated with the use of statins, ACE inhibitors/ARBs, and a trend for a lower event rate with the use of β-blockers (hazard ratio, 0.86 [95% CI, 0.74–1.01]), while dual antiplatelet treatment was not associated with a lower event rate. [60]

The results of the MINOCA-BAT trial (NCT03686696), randomizing 3500 MINOCA patients to β-blocker and ACEi or ARB versus placebo, will shed additional light on the topic, and are expected in 2025 [61].

In patients with suspected vasospastic angina, calcium-channel blockers and nitrates should be preferred as second-line treatments; nicorandil and cilostazol can also be considered as potential options [62].

On the contrary, in patients with suspect plaque erosion or calcific nodules, potentially triggering the formation of thrombus, the use of antithrombotic agents should be encouraged, based on the results of the EROSION (Effective Anti-Thrombotic Therapy Without Stenting: Intravascular Optical Coherence Tomography-Based Management in Plaque Erosion) study [63].

Thus, the identification of the underlying mechanism and the tailoring of pharmacological therapy appears to be vital for the appropriate management of these patients.

Therefore, a “full” physiology approach, including a complete assessment of epicardial and microvascular circulation, should be advocated in order to relieve symptoms and prevent ischemia, thus improving the outcomes of these patients. Nevertheless, further studies are certainly warranted to define the most appropriate indexes and the prognostic implications of a physiology-based strategy.

## Figures and Tables

**Table 1:** Technical features, advantages, and pitfalls of the different indexes for the assessment of coronary physiology.

	FFR	iFR	CFR	IMR
Cut-off	≤0.80	≤0.90	≤2.0	>25 U
Timing of acquisition	Averaged (5 cycles)	Single beat	Averaged (3 cycles)	Averaged (3 cycles)
Stressing agent	Adenosine, papaverine, other	-	Adenosine, papaverine, other	Adenosine, papaverine, other
Epicardial stenosis	+	+	+	-
Microcirculation assessment	-	+	+	+
Validation in ACS	-	+	-	-
Tandem lesions	-	+	+	+
Side branch correction	-	+	+	+
Co-registration	-	+	-	-
Low-flow condition sensitivity	+	-	-	-
Wire drift sensitivity	+	-	+	-

ACS = acute coronary syndrome; FFR = fractional flow reserve; iFR = instant wave-free flow reserve; CFR = coronary flow reserve; IMR = index of microvascular resistance; U = units.
